# Primary malignant tumours and malignant transformation of cysts in the retrorectal space: MRI diagnosis and treatment outcomes

**DOI:** 10.1093/gastro/goac048

**Published:** 2022-09-20

**Authors:** Jing Gong, Yumeng Xu, Yan Zhang, Lichao Qiao, Haixia Xu, Ping Zhu, Bolin Yang

**Affiliations:** Department of Colorectal Surgery, Jiangsu Province Hospital of Chinese Medicine, Affiliated Hospital of Nanjing University of Chinese Medicine, Nanjing, Jiangsu, P. R. China; First Clinical Medical College, Nanjing University of Chinese Medicine, Nanjing, Jiangsu, P. R. China; Department of Colorectal Surgery, Jiangsu Province Hospital of Chinese Medicine, Affiliated Hospital of Nanjing University of Chinese Medicine, Nanjing, Jiangsu, P. R. China; First Clinical Medical College, Nanjing University of Chinese Medicine, Nanjing, Jiangsu, P. R. China; Department of Colorectal Surgery, Jiangsu Province Hospital of Chinese Medicine, Affiliated Hospital of Nanjing University of Chinese Medicine, Nanjing, Jiangsu, P. R. China; First Clinical Medical College, Nanjing University of Chinese Medicine, Nanjing, Jiangsu, P. R. China; Department of Colorectal Surgery, Jiangsu Province Hospital of Chinese Medicine, Affiliated Hospital of Nanjing University of Chinese Medicine, Nanjing, Jiangsu, P. R. China; First Clinical Medical College, Nanjing University of Chinese Medicine, Nanjing, Jiangsu, P. R. China; Department of Colorectal Surgery, Jiangsu Province Hospital of Chinese Medicine, Affiliated Hospital of Nanjing University of Chinese Medicine, Nanjing, Jiangsu, P. R. China; First Clinical Medical College, Nanjing University of Chinese Medicine, Nanjing, Jiangsu, P. R. China; Department of Colorectal Surgery, Jiangsu Province Hospital of Chinese Medicine, Affiliated Hospital of Nanjing University of Chinese Medicine, Nanjing, Jiangsu, P. R. China; Department of Colorectal Surgery, Jiangsu Province Hospital of Chinese Medicine, Affiliated Hospital of Nanjing University of Chinese Medicine, Nanjing, Jiangsu, P. R. China

**Keywords:** retrorectal tumour, malignant transformation, clinical features, treatment, prognosis

## Abstract

**Background:**

There are no clear guidelines for the diagnosis and treatment of malignant retrorectal tumours. The purpose of this study was to increase preoperative diagnostic knowledge and to describe the outcomes of treatment for these patients.

**Methods:**

This retrospective study was conducted on patients who underwent complete retrorectal tumour resection between May 2006 and July 2018, and had confirmed post-operative pathology reports. Demographic and clinical data (including imaging, perioperative, pathological, and prognostic data) were collected and analysed.

**Results:**

Malignant lesions were identified in 15 (9 [60%], female) patients. The median age of the patients was 59 years (range, 34–72 years). Primary malignant tumours were identified in seven patients with solid tumours, in which gastrointestinal stromal tumours accounted for 71.4% (five of seven) and the remainder were chordoma or mucinous adenocarcinoma. Malignant transformation of cysts occurred in another eight patients with heterogeneous tumours, while histopathological features were present in 75% (six of eight) of patients with mucinous adenocarcinoma, and the remainder were squamous-cell carcinoma or neuroendocrine tumour (Grade 2). The malignant characteristics of the solid portions observed using magnetic resonance imaging (MRI) were as follows: the cyst wall of the tumour was irregularly thickened; the surface was convex or lobed; the solid tumour had no capsule, or the capsule was destroyed; and the surface had a gyrus-like morphology. At a median follow-up time of 52 months (range, 13–100 months), the overall recurrence-free survival rate was 40.0% and the survival rate was 46.7%.

**Conclusion:**

Some MRI features can be used to distinguish malignant retrorectal tumours from benign retrorectal tumours. The survival rate of patients with malignant retrorectal tumours is poor.

## Introduction

Tumours located in the retrorectal space comprise a rare heterogeneous group with an estimated incidence ranging from 1 in every 40,000 to 63,000 hospital admissions per year [1, 2]. The occurrence of malignant tumours and malignant transformation of cysts is reported to be extremely low. In a retrospective study published in 2016, only 514 cases of malignant tumours were included from 341 articles, most of which (449 of 514, 87.4%) were primary malignant tumours and a few of which (65 of 514, 12.6%) were malignant transformation of cysts [3]. There has been a lack of large sample data and studies conducted on small sample sizes have made it difficult to establish a standard protocol for the diagnosis and treatment of these types of tumours.

In recent years, imaging examination has been shown to be necessary for the diagnosis of retrorectal tumours, with computed tomography (CT) and magnetic resonance imaging (MRI) able to effectively differentiate benign from malignant tumours [4–6]. It was reported that MRI (17 of 18) tended to have better performance in differentiating between benign and malignant tumours than CT (9 of 14) (94% vs 64%, *P *=* *0.06) [7]. MRI can guide surgeons to select the most appropriate surgical approach because it can delineate peritumoural planes and determine local invasion (rectum and/or sacrum) and nerve involvement [8]. Preoperative biopsies that do not affect therapy protocols are unnecessary when the tumour can be completely resected [9]. In cases of cystic lesions, biopsy is contraindicated due to the potential risk of infection; biopsy of sacral meningocele increases the risk of meningitis [7]. If biopsy is necessary, the needle tract should be resected en bloc with the specimen to decrease the risk of seeding and local recurrence. Given the potential for malignant lesions, surgery is the primary treatment option available for resectable retrorectal tumours [10].

Recurrence is associated with tumour biology, incomplete resection with positive margins, breach of the tumour capsule, and tumour spillage [5, 10, 11]. Patients with malignant retrorectal tumours usually experience a higher rate of recurrence and have poorer outcomes than those with benign retrorectal tumours with an overall 5-year survival rate reported to range from 51% to 75% [5, 7, 12, 13]. However, due to the availability of only a few reports and the small number of samples included, the results of the comparison between primary malignant tumours and malignant transformation of cysts in the retrorectal space cannot be confirmed and the exact prognosis is unclear.

This study aimed to increase knowledge about the preoperative diagnosis of primary malignant tumours and malignant transformation of cysts in the retrorectal space, and to evaluate the follow-up outcomes of patients treated at our institution.

## Methods

### Patients

A retrospective study was conducted to include the medical charts of discharged patients diagnosed with retrorectal tumours at Jiangsu Province Hospital of Chinese Medicine (Nanjing, China) between May 2006 and July 2018 obtained from the Electronic Medical Records System. After we reviewed the medical records on all retrorectal tumours, patients with complete tumour resection and post-operative pathologically confirmed malignancies were further analysed. The tumour should be resected entirely while keeping the resection margin clear during the operation. Primary malignant tumours are pathologically defined as malignant solid tumours; the malignant transformation of cysts is pathologically confirmed as developing cysts with malignant elements in local tissue.

### Data collection and follow-up

Data extracted from patient charts included demographic characteristics, clinical presentations, diagnostic evaluations (including imaging information, operative approaches, and the histological types of the tumours), interval to recurrence, and survival time after surgery. The follow-up was conducted at the clinic or through telephone interviews. All patients were required to return to our hospital for follow-up visits every 3–6 months for a period of 2 years, and then every 6 months during the subsequent 3-year period and once per year thereafter. Follow-up ceased if the patients died or as a result of data censoring.

### Statistical analysis

We used SPSS statistical software, version 25.0, for all statistical analyses (Chicago, IL, USA). Descriptive statistics included medians and ranges for non-normally distributed quantitative variables, and numbers and percentages for categorical variables. Differences in categorical variables between groups were tested using the χ^2^ test or Fisher’s exact test, while differences between quantitative variables were compared using the Mann–Whitney *U* test. Kaplan–Meier estimates were used for recurrence-free survival differences and survival analysis. The one-sided *t*-test was performed, where statistical significance was defined as *P *<* *0.05.

## Results

### Patient characteristics

**Figure 1.**
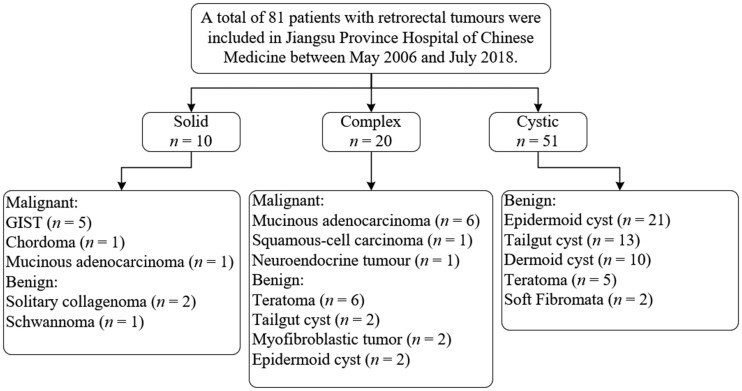
Flowchart showing the inclusion process of patients. GIST, gastrointestinal stromal tumour.

A total of 81 patients with retrorectal tumours were identified, of whom 15 received a post-operative pathological diagnosis of malignancy (Figure 1). The malignant patients were of a median age of 59 years old (range, 34–72 years), including seven patients with primary malignant tumours and eight patients with malignant transformation of cysts. Malignant transformation of cysts was more likely to occur in women than in men (F:M = 7:1), while primary malignant tumours showed the opposite trend (F:M = 2:5). The median interval from the onset of symptoms to the diagnosis of malignant tumours was 12 months (range, 1–720 months). Gastrointestinal stromal tumours (GISTs) were mostly observed in primary malignant tumours in our study (five of seven, 71.4%), followed by chordoma (one of seven, 14.3%) and mucinous adenocarcinoma (one of seven, 14.3%). The pathology of the malignant transformation of cysts included six mucinous adenocarcinomas, one squamous-cell carcinoma and one neuroendocrine tumour (Grade 2), respectively. Tailgut cysts had a greater likelihood of developing into malignant tumours (4 of 19, 21.1%), followed by dermoid cysts (1 of 11, 9.1%), teratomas (1 of 12, 8.3%), and epidermoid cysts (2 of 25, 8.0%). The most frequent symptoms among patients with malignant retrorectal tumours were pain (6 of 15, 40.0%), constipation (3 of 15, 20.0%), tenesmus (2 of 15, 13.3%), perianal suppuration (2 of 15, 13.3%), lower limb dysfunction (1 of 15, 6.7%), and extra-rectal masses (4 of 15, 26.7%), which were found through digital rectal examination. Digital rectal examination of palpable extra-rectal masses was the most commonly used diagnostic method (30 of 66, 45.5%). The detailed demographics and clinical characteristics of the patients are summarized and presented in Tables 1 and 2.

**Table 1.** Demographics and clinical characteristics of the patients with malignant transformation cysts in the retrorectal tumours

Patient	Gender	Age at diagnosis of malignant transformation, years	Interval from the onset of symptoms to degeneration, months	Symptoms leading to surgery	Mass nature	Surgical approach	Histopathology		Adjuvant therapy	Relapse time after surgery, months	Recurrence location	Time of death after surgery, months
							Benign ingredient	Malignant ingredient				
1	F	34	30	Persistent pain	Complex	PA (coccygectomy)	Tailgut cyst	Mucinous adenocarcinoma	NA	27	Local, distant	52
2	M	65	216	Extra-rectal mass	Complex	PA (coccygectomy)	Epidermoid cyst	Squamous-cell carcinoma	No	20	Local, distant	21
3	F	60	12	Perianal suppuration	Complex	PA (coccygectomy)	Tailgut cyst	Mucinous adenocarcinoma	FOLFOX-6 and DT34 Gy/17f	31	Local	65
4	F	66	720	Extra-rectal mass and perianal suppuration	Complex	PA (coccygectomy)	Tailgut cyst	Mucinous adenocarcinoma	No	21	Local	28
5	F	52	1	Tenesmus	Complex	PA	Teratoma	Mucinous adenocarcinoma	NA	23	Local, distant	57
6	F	44	12	Persistent mass	Complex	PA	Dermoid cyst	Mucinous adenocarcinoma	No	No	No	No
7	F	44	2	Persistent pain and constipation	Complex	PA	Epidermoid cyst	Neuroendocrine tumour (Grade 2)	DT50 Gy/25f	57	Local	No
8	F	66	1	Tenesmus	Complex	PA (coccygectomy)	Tailgut cyst	Mucinous adenocarcinoma	No	No	No	No

F, female; M, male; PA, posterior approach; NA, not available.

**Table 2.** Demographics and clinical characteristics of the patients with primary malignant retrorectal tumours

Patient	Gender	Age at diagnosis of malignant tumour, years	Symptoms leading to surgery	Mass nature	Surgical approach	Histopathology	Adjuvant therapy	Relapse time after surgery, months	Recurrence location	Time of death after surgery, months
9	F	66	Lower limb dysfunction	Solid	CA (coccygectomy and sacrectomy)	Chordoma	No	13	Local	13
10	M	39	Persistent pain and constipation	Solid	PA (coccygectomy)	GIST	No	No	No	No
11	M	59	Constipation	Solid	PA (coccygectomy)	GIST	Imatinib	No	No	No
12	M	45	Persistent pain	Solid	PA (coccygectomy)	GIST	Imatinib	No	No	No
13	M	56	Persistent pain	Solid	AA	GIST	No	3	Local	15
14	M	61	Persistent pain	Solid	PA	GIST	No	No	No	No
15	F	72	Persistent mass	Solid	PA (coccygectomy)	Mucinous adenocarcinoma	Cetuximab	36	Distant	87

F, female; M, male; PA, posterior approach; CA, combined approach; AA, abdominal approach; GIST, gastrointestinal stromal tumour.

### MRI features of the malignant tumours

The solid portion, such as enhancement, necrosis, or haemorrhage, in the tumours presented as an intermediate or slightly higher signal on T2-weighted images. Solid tumours were composed of >80% solid elements and appeared in seven patients with malignant tumours and three patients with benign tumours. The diagnosis of a cystic tumour was made when the lesion displayed >80% cystic elements and this diagnosis was found in 51 patients with benign tumours. The remainder were classified as heterogeneous tumours and were found in 8 patients with malignant tumours and in 12 patients with benign tumours. All malignant tumours included in this study had solid tissues, while 15 benign lesions (22.7%, *n *=* *66) had solid components, including 12 complex tumours and 3 solid tumours. The sensitivity and specificity for malignancy based on the presence of solid tissue were 100% (15 of 15) and 77.3% (51 of 66), respectively.

**Figure 2.**
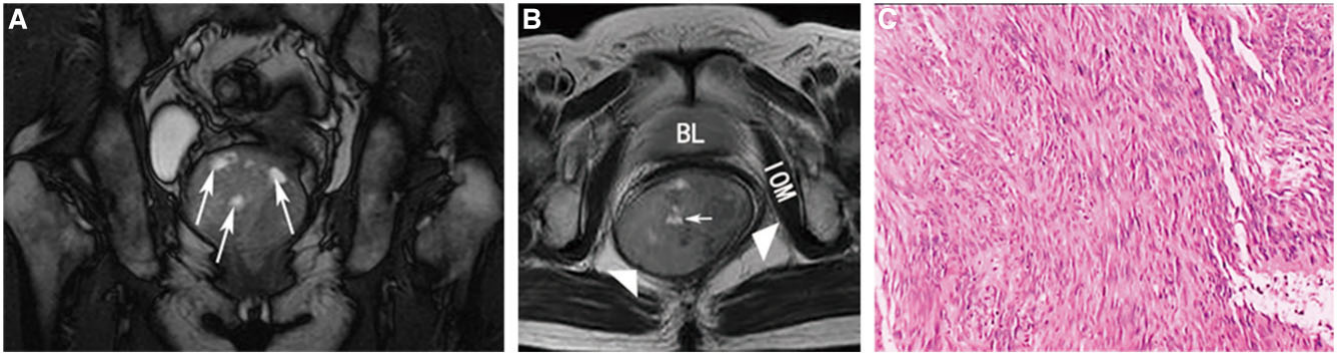
Presacral gastrointestinal stromal tumours in a 51-year-old woman. Images are from Bolin Yang [23]. (A) and (B) A large mass with a low-intensity signal and some necrosis that exhibited hyperintensity (white arrows) on T2-weighted magnetic resonance images. The complete capsule exhibited a low-intensity signal (white triangles). (C) Histopathology shows spindle cells and cytoplasmic vacuoles (haematoxylin and eosin staining, ×100). BL, bladder; IOM, internal obturator muscle.

**Figure 3.**
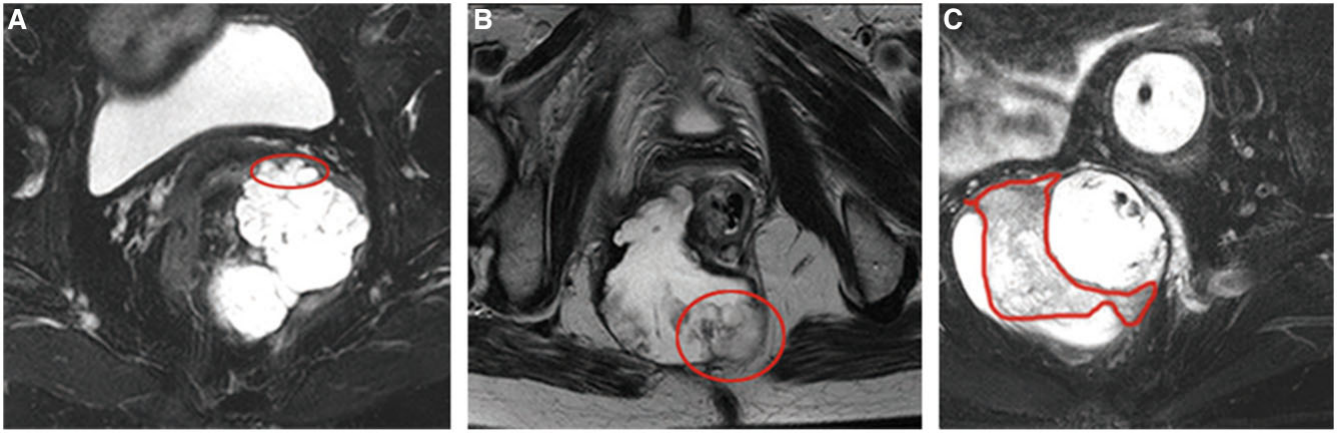
Magnetic resonance imaging (MRI) features of the solid portions of malignant tumours. (A) Mucin forms a major component of primary mucinous adenocarcinoma. The tumour has no capsule, showing a high signal intensity on fat-suppressed T2-weighted MR images. (B) The wall of the epidermoid cyst with components of a squamous-cell carcinoma is damaged and irregular, and the lobulated contour has surface projections, which exhibit slightly higher signal intensity on T2-weighted images. (C) There are intermediate-intensity portions of mucinous adenocarcinoma arising from the tailgut cyst on fat-suppressed T2-weighted MR images. The malignant components are circled in red.

**Figure 4.**
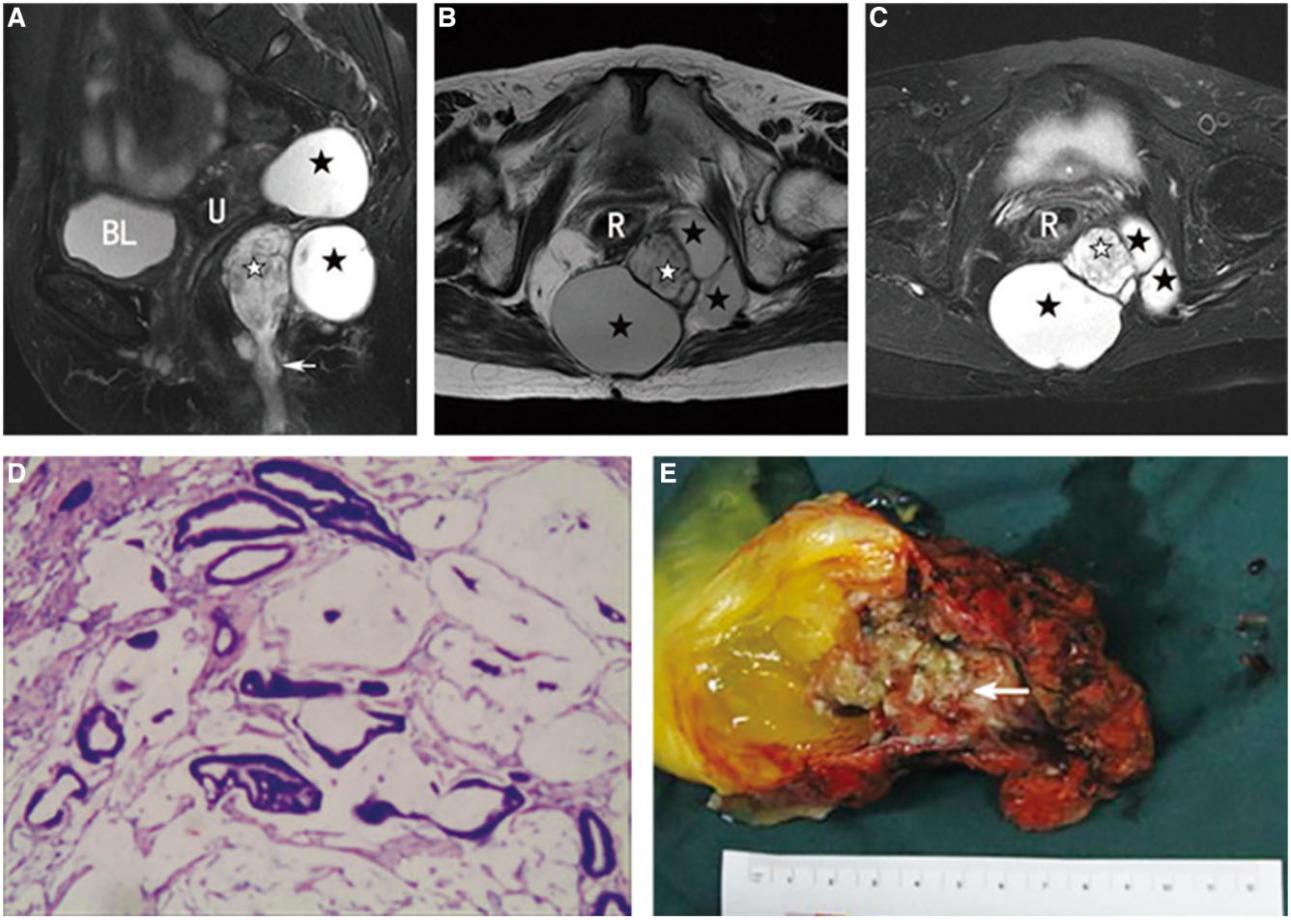
Clinical data from a 66-year-old female patient with inhomogeneous retrorectal tumours. The tailgut cysts were present in her for 60 years and she had undergone malignant transformation. (A) Sagittal T2-weighted fat suppression magnetic resonance (MR) image. The early drainage channel leading to the anal margin can be observed at the far end (white arrow). (B) Axial T2-weighted MR image. (C) Axial T2-weighted fat suppression MR image. (D) Histology confirmed that the solid tissues in the cysts were mucinous adenocarcinoma (haematoxylin and eosin staining, ×100). (E) Solid tumours growing in the cavity are seen in the cysts (white arrow). Homogeneous high intensity indicates cystic components (dark asterisks), while inhomogeneous high intensity indicates mixed components (white asterisks), which were pathologically confirmed as mucinous adenocarcinomas. BL, bladder; U, uterus; R, rectum.

**Figure 5.**
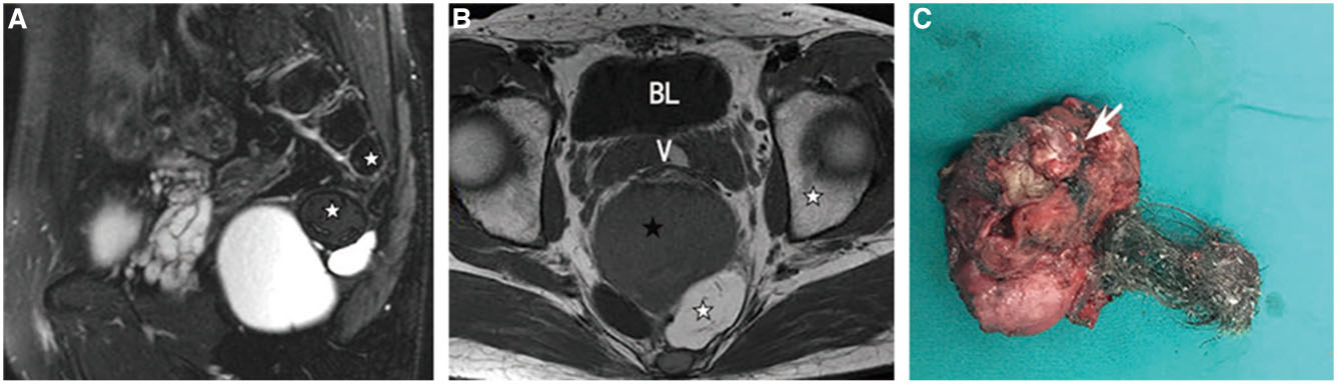
Magnetic resonance imaging of a patient with a teratoma. (A) Sagittal T2-weighted fat suppression image. (B) Axial T1-weighted image. (C) The solid components in the tumour are bony (white arrow). Homogeneous low intensity (T1W1) indicates cystic components (dark asterisks), while low intensity (T2W1-FS) or inhomogeneous slightly high intensity (T1W1) indicates solid components (white asterisks), which were confirmed as bony or calcified. BL, bladder; V, seminal vesicle.

The MRI characteristics of the solid portions of the malignant tumours are shown in Figures 2–4. GISTs exhibited a low-intensity signal, while some necrosis presented as hyperintensity on T2-weighted MR images (Figure 2). The complete capsule showed a low-intensity signal. Primary mucinous adenocarcinoma displayed a hyperintense signal on fat-suppressed T2-weighted images (Figure 3A). It had no capsule and the surface resembled the shape of brain circuits, while the rim was irregular with surface projections. The damaged capsule showed a signal of moderate intensity on T2-weighted images, while the rim was irregular with surface projection in the epidermoid cyst with components of squamous-cell carcinoma (Figure 3B). Mucinous adenocarcinoma arising from the tailgut cyst manifested as intermediate tissue shadows on fat-suppressed T2-weighted images (Figure 3C). Heterogeneous tumours were found in patients with malignant transformation of cysts with components of mucinous adenocarcinoma. The malignant portion exhibited high signal intensity and produced mesh-like enhanced areas on the fat-suppressed T2-weighted images. The cystic portion displayed hyperintensity on T2-weighted images with regular borders (Figure 4). In benign tumours, the solid tissues exhibited a smooth or lobular contour without surface projections. The solid components of mature teratoma, defined as bone, exhibited a slightly high intensity on T1-weighted images and low intensity on T2-weighted fat-suppressed images (Figure 5). The internal signalling characteristics of the tumours were homogeneous and the margin was found to be well defined.

### Treatment

**Figure 6.**
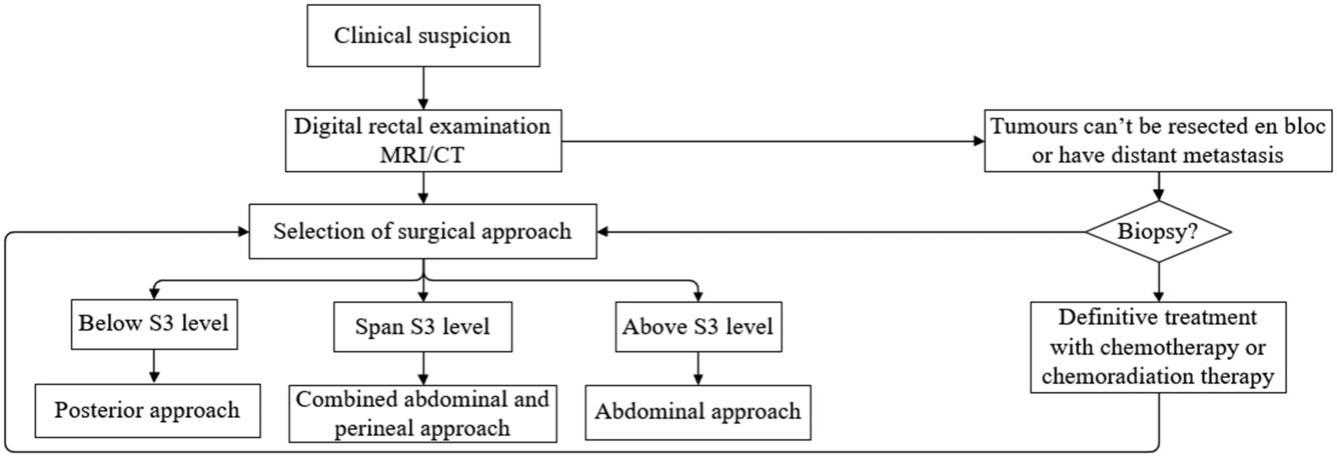
Flowchart showing the method of surgical selection.

The flow chart illustrates the algorithm used for surgical selection (Figure 6). The definitive method of treatment was surgery and aimed to achieve en bloc resection. Among the cases, 13 patients received R0 resection, while 2 patients received R1 resection (Patients 3 and 7). Thirteen patients underwent the posterior approach, which is suitable for tumours below the third sacral body (S3), while the other two patients underwent the combined anterior and posterior approach for tumours that extended to both the proximal and distal areas of the third sacral body. Adjuvant therapies were applied based on the type of tumour as identified through pathology. The patients who received R1 resection all received adjuvant therapies. A patient with primary mucinous adenocarcinoma received cetuximab, a patient with a tailgut cyst who developed malignant transformation of mucinous adenocarcinoma received FOLFOX-6 and DT34 Gy/17f, another patient with neuroendocrine tumour arising in an epidermoid cyst was treated with DT50 Gy/25f, while two patients with GISTs received imatinib. A patient with tailgut cyst and another patient with a teratoma who developed malignant transformation of mucinous adenocarcinoma were transferred to another hospital for treatment and did not provide us with their treatment plans. None of the other patients received adjuvant therapies.

### Outcomes of the long-term follow-up

The median length of post-operative follow-up in patients with malignant tumours was 52 months (range, 13–100 months). Eight (53.3%) patients developed local recurrence, of whom three patients developed distant recurrence as well, while one patient showed only distant recurrence.

Among the 15 patients, 3 of the 5 patients who received adjuvant therapies showed recurrence at 31, 57, and 36 months. Two patients died at 65 and 87 months after surgery. Four of the eight patients who did not receive adjuvant therapies showed recurrence at 20, 21, 13, and 3 months, and died at 21, 28, 13, and 15 months. Two patients who did not provide their treatment plans showed recurrence at 27 and 23 months, and died at 52 and 57 months, respectively. The recurrence rates of tumours with malignant transformation of cysts and primary malignant tumours were 75.0% (6 of 8) and 42.9% (3 of 7), respectively. The death ratios of patients with tumours with malignant transformation of cysts and primary malignant tumours were 62.5% (five of eight) and 42.9% (three of seven), respectively. The overall recurrence-free rate and the survival rate were similar between patients diagnosed with primary malignant tumours and those who developed malignant transformation of cysts (log-rank test: *P *=* *0.36 and *P *=* *0.33, respectively).

## Discussion

The majority of adult retrorectal tumours are benign but might undergo malignant changes. A retrospective study reported that malignant tumours accounted for 18% of all retrorectal tumours [14]. This study was conducted on a sample size of 81 patients with retrorectal tumours at a single centre and the incidence of malignancy was 18.5% (15 of 81). Malignant tumours were found to be more prevalent in elderly patients [15]. The median age of patients with malignant tumours was 59 years. Developmental cysts with solid components were the most likely to undergo malignant transformation and the malignant tumour type was predominantly mucinous adenocarcinoma. In our dataset, 21.1% (4 of 19) of patients with a large component being a tailgut cyst were more likely to undergo malignant transformation and the rate was much higher than previously reported (2%–13%) [16, 17]. However, this finding is consistent with the results of a previously published systematic review (26.6%) [18].

MRI can be used to determine whether preoperative biopsy is necessary. Biopsy is not considered if the retrorectal tumours can be completely removed [19, 20]. MRI is also useful for distinguishing between benign and malignant entities, although advanced imaging technology alone should not be relied upon for an exact diagnosis [4, 5, 14]. MRI can provide better tissue characterization to reveal the loss of defined cyst margins and the involvement of neighbouring structures in cases of malignant transformation due to its high contrast resolution between different tissue compartments compared with CT scans [21, 22]. The most important finding was that malignancy could be observed as a solid tissue component in lesions that exhibited intermediate to high signal intensity on T2-weighted MR images. The solid tissue component could also exhibit high signal intensity on fat-suppressed T2-weighted images due to the presence of mucinous materials, high protein content, or haemorrhage in the tumour. The internal structures of the cystic lesions (unilocularity, multilocularity, debris, septa, and wall thickening) were not predictive of its benign or malignant status [21].

The characteristic MRI observations of tumours with solid tissues were presented in a previous report [23]. The solid parts of the tumours exhibit low signal intensity on T1-weighted MR images, intermediate to high signal intensity on T2-weighted MR images, and enhancement after the administration of gadolinium. The significantly high signal observed on T2-weighted MRI should be considered a feature that strongly indicates the diagnosis of GIST. Another type of malignant solid tumour, chordoma, shows obvious osteolytic damage to adjacent bones. The tumour is mainly composed of mucus interstitium with a long T2 relaxation time and mucus-secreting droplet tumour cells. The obvious high signal intensity on T2W1 images reflects the histological characteristics of chordoma. Tumours can exhibit solid components but might not be malignant. Teratomas with solid tissues commonly occur in neonates and infants, and are usually benign, but those found in adults can undergo malignant transformation if left untreated [20, 24]. We can differentiate between malignant and benign tumours using the following characteristics shown on MR imaging: the cyst wall is irregularly thickened and the surface is convex or lobed. A solid tumour has no capsule, the surface has a gyrus-like morphology, or the capsule has been destroyed. A heterogeneous tumour with an irregular margin is usually malignant, while a cystic tumour with solid components has a smooth, well-circumscribed margin; the tumour is benign if no features of invasion or enhancement are visible following the administration of gadolinium.

Due to the limited operating space, we recommend that the sacrococcyx should be removed during surgery for R0 resection. It is also necessary to conduct a coccygectomy or sacrectomy if the mass is adherent to the coccyx or sacrum [25]. Although not statistically significant, patients with cysts who developed malignant transformation had a higher recurrence rate and death rate than those with primary malignant tumours (75.0% vs 42.9%, 62.5% vs 42.9%). The overall recurrence-free survival rate was 40.0% and the survival rate was 46.7% for all malignant tumour patients. Patients with malignant retrorectal tumours showed a high rate of recurrence and had poor prognoses. Therefore, both neoadjuvant and adjuvant treatments, including radiation, might need to be considered, especially in patients with squamous-cell carcinomas or mucinous adenocarcinomas.

The limitations of our study include the retrospective nature and the inherent bias due to its being based on data obtained from a single institution. Since there is currently no standard method of treatment for malignant tumours, these patients received therapeutic strategies that lacked uniformity. Chemotherapy or radiotherapy was not used on every patient with a malignancy, which might have been an error in treatment. Subsequently, these cases might be subject to selection bias or other unknown factors. In addition, it might not be possible to generalize these results due to the rarity of retrorectal tumours.

## Conclusions

MRI can be used to indicate preoperative malignancy through the identification of solid lesions. It is important to closely follow patients with developmental cysts that have solid tissue components due to their malignant potential. Regardless of whether radiochemotherapy was administered, the long-term survival rate of patients after surgery was poor. Patients with cysts who develop malignant transformation tend to have a higher recurrence rate and mortality rate than those with primary malignant tumours.

## Authors’ Contributions

J.G. and Y.X. extracted the data and drafted the manuscript. Y.Z., L.Q., and H.X. analysed and interpreted the data. P.Z. and B.Y. revised the manuscript critically for important intellectual content. All authors read and approved the final manuscript.

## Funding

This work was supported by the developing Program for High-level Academic Talent in Jiangsu Hospital of TCM (Grant No. y2021rc27) and Phase III Project Funded by the Priority Academic Program Development of Jiangsu Higher Education Institutions (Grant No. ZYX03KF034).
